# THEME: “Vaccines and Vaccine Adjuvants/Immunomodulators for Infectious Diseases”

**DOI:** 10.3390/vaccines11020383

**Published:** 2023-02-07

**Authors:** Sruthi Vijaya Retnakumar, Srinivasa Reddy Bonam, Haitao Hu, Jagadeesh Bayry

**Affiliations:** 1 Institut National de la Santé et de la Recherche Médicale, Centre de Recherche des Cordeliers, Sorbonne Université, Université de Paris, 75006 Paris, France; 2 Department of Microbiology and Immunology, University of Texas Medical Branch, Galveston, TX 77555, USA; 3 Institute for Human Infections & Immunity, Sealy Institute for Vaccine Sciences, University of Texas Medical Branch, Galveston, TX 77555, USA; 4 Department of Biological Sciences & Engineering, Indian Institute of Technology Palakkad, Palakkad 678623, India

The discovery of vaccines has enabled the successful prevention of many deadly infectious diseases, decreased the overall mortality rate, and improved life expectancy worldwide [1]. Despite a long history of advancements in vaccine research, several newly emerging and re-emerging pathogens continue to present threats to the global population, and vaccine immunologists are experiencing more challenges than ever before.

The basic principle of vaccination is grounded in the concept of ‘immunological memory’, whereby the immune system can induce a more rapid and effective response to a previously encountered foreign antigen via pre-existing clonally expanded memory lymphocytes. A vaccine should, therefore, consist of a biological agent that resembles the disease-causing pathogen, which is able to induce an adequate immune response without causing pathogenicity in the host [2]. The history of vaccination begins with Edward Jenner’s successful experiment, wherein he used the crude isolate of cowpox virus to prevent smallpox in humans. The concept of live-attenuated pathogens as non-virulent vaccine agents was deliberately demonstrated first by Luis Pasteur in his observations of chicken cholera, which led to the development of vaccines against anthrax and rabies. Following this, the concept of inactivated vaccines emerged from the fact that the pathogen does not need to be alive to mimic the desired immune response, which resulted in the discovery of safe and widespread use of a series of killed pathogens for preventing the large number of diseases, including typhoid, cholera, polio, tuberculosis, yellow fever, and others [3]. The advancements in molecular biology and genetics have resulted in the advent of various innovative platforms for vaccine development in later years, utilizing several subcellular components of the pathogen, rather than the whole organism. Protein subunit vaccines consisting of critical recombinant soluble protein antigens are capable of inducing an immune response, whereas virus-like particle vaccines are composed of a viral envelope devoid of the genetic material. The most recent addition to this repertoire are DNA/RNA-based vaccines, which utilize only the genetic material from the pathogens. Each of these platforms stimulates immune responses through distinct mechanisms due to which the efficacy and safety of these vaccine platforms vary across different pathogens [4].

Regardless of the safety and economic viability demonstrated by subunit vaccines, they are consistently weakly immunogenic, mainly due to the absence of complete repertoire of pathogen-associated molecular patterns (PAMPs) possessed by whole pathogens, which are critical in activating the host’s innate immune system [2]. This resulted in the development of adjuvants, which are substances added to the vaccines to enhance or prolong the resulting immune response. In addition to economizing the use of recombinant subunit vaccines, adjuvants can also minimize the doses of inactivated vaccines. Moreover, adjuvants exhibit the capacity to stabilize the antigens by protecting them from degradation. Based on their mode of action, some of the adjuvants can be classified as immunopotentiators (flagellin, muramyl dipeptide [MDP], and cytosine phosphoguanosine [CpG]), which stimulate the innate immune system by interacting with pattern recognition receptors and, hence, activate various downstream immune signaling pathways. In contrast, another class of compounds, including alum and oil-based adjuvants, acts as a delivery system that carries antigens to immune cells to promote antigen-presentation by major histocompatibility complex (MHC) molecules. Finally, adjuvants are known to determine the type of adaptive immune response generated in response to vaccination [5,6]. Hence, in addition to the general population, the design and use of appropriate adjuvants are particularly important in older adults due to the phenomenon of immunosenescence characterized by age-related decline in the immune response against vaccines [7,8], as well as in immunocompromised individuals having a weaker immune system [9].

The first approved, and still the most widely used adjuvant, is alum, and only five more adjuvants (MF59, adjuvant systems (AS)01, AS04, AS03, and CpG) have been approved for clinical use in recent years. The huge gaps in vaccine adjuvant research are clearly visible, as the precise molecular mechanisms behind these adjuvants have not been elucidated completely [10]. Despite the safety and ease of manufacturing processes, the immune response induced by alum is biased towards type 2 immunity, which favors mainly humoral antibody responses [11]. The best vaccine protection would be elicited by a combination of strong humoral and cellular immune responses by mixed T helper and T follicular responses, and ongoing efforts focus on developing combined adjuvant formulations that can attain this goal.

A better understanding of the innate and adaptive immune systems and the development of system biology approaches that can identify the global molecular patterns at the single-cell level has provided new momentum to the field of vaccine research, facilitating the fast-paced development of novel vaccine platforms and mechanism-based immunomodulatory adjuvants [10]. This special issue on vaccines is concerned with expounding upon interesting recent developments in the field of vaccine and vaccine adjuvant research. Our last special issue, “Vaccines and Vaccine Adjuvants/Immunomodulators for Infectious Diseases”, had great success with the publication of six review articles. In addition, the 2nd edition of this special issue continues to disseminate more novel discoveries in the field with the contribution of two original research articles so far. We have briefly summarized those articles here.

The COVID-19 pandemic has imparted devastating effects on the health of people and heavily disrupted the socioeconomic status of countries around the globe. However, as a boon in disguise, the pandemic has provided a fertile ground for groundbreaking developments in vaccine research. The situation challenged us with a need for developing vaccines at a pressing speed and revealed the urgent need to develop tools and design policies for better preparedness in facing pandemic outbreaks. Two articles from Kamareddine and team extensively reviewed the challenges and safety concerns faced during the COVID-19 vaccination rollout and proposals to overcome these problems [12,13]. The first article is a comprehensive review of the literature of various vaccine platforms being used for the development of COVID-19 vaccines that were in the pipeline since the start of the pandemic or have been approved for use. The authors furnish detailed descriptions of each platform, emphasizing their strengths or limitations and identifying potential opportunities and risks associated with them. In addition to the widespread and extensive use of the existing vaccine platforms with promising technological advancements, one of the greatest achievements attained by the pandemic was the approval of the first mRNA-based vaccines for human use by Pfizer-BioNTech and Moderna. The impressive efficacy demonstrated by the newly developed mRNA vaccines appears promising, and the future of mRNA vaccines may be exceedingly gleaming with regard to their cost-effective manufacturing and rapid development. Nevertheless, owing to the recent discovery, the long-term side effects of this vaccine platform are yet to be determined. Strategies combining multiple vaccine platforms are also in the pipeline, which can overcome the disadvantages of the individual platforms and impart more potent protective immune responses [12].

In addition to the technical challenges in improving the safety and efficacy of vaccines, the COVID-19 vaccine deployment was affected by various socioeconomic factors, which were the central points of discussion in the second article [13]. The data remain scarce regarding the long-term safety of these vaccines, the duration of immunological memory, and the ability of currently available vaccines to protect from the newly emerging variants. Another major hurdle is the conduct of clinical trials incorporating all categories, especially the vulnerable populations comprising the elderly and immunocompromised individuals. The obvious differences in vaccine immunogenicity, important safety concerns, and potential interaction of vaccines with other medications among these categories (for example, cancer patients undergoing various treatment modalities [14]) require urgent attention. From a socioeconomic point of view, ensuring equitable access to vaccines regardless of the purchasing power of each country is a key challenge, particularly for low-middle-income countries. The authors also emphasize the necessity to create awareness and trust among people to avoid vaccine hesitancy, which can significantly impede the efforts to establish herd immunity. Altogether, in order to fully benefit from the scientific and technological advances in vaccine research, policies should be implemented to ensure fair access to vaccines, and measures should be taken to educate and control the spread of misinformation among the public [13].

Another field of vaccine research that has immensely benefited from the COVID-19 pandemic is the development of human viral vector vaccines. Recombinant viral vectors created from certain viruses by removing nonessential genes serve as safe antigen carrier vehicles for a target antigen. In general, live-attenuated vaccines are known to strongly activate both cellular and humoral immune responses by presenting antigens via class I or class II MHC molecules. However, the requirement of high containment laboratories, the risk of infection or pathogen reversion to wild type, etc. limit their utilization in several highly infectious pathogens. Live viral vector vaccines behave similarly to live-attenuated vaccines in terms of their immunogenicity while overcoming their safety concerns. Furthermore, recombinant viral vectors also have the capacity to present vaccine antigens in a proper conformation more efficiently than subunit vaccines [15]. Vilela et al. discussed the characteristics of one such viral vector based on avian orthoavulavirus type-1 (AOaV-1), which is widely used in poultry and animals (sheep, dogs, cats, pigs, horses, and camels). Recent pre-clinical evidence supports the utilization of this vector for human viruses, including influenza and coronaviruses [16]. The COVID-19 era witnessed the popularization of viral vectors for human use with three adenoviral vector-based vaccines licensed for SARS-CoV-2. The review by Daian e Silva and colleagues gave a detailed account of different viral vectors that have been used for SARS-CoV-2 vaccine development [17]. The authors compared the advantages and disadvantages of each of these vectors and the importance of heterologous prime-boost vaccination to avoid the interference of the primary immune response to the vector with the subsequent doses. A major disadvantage of vectored vaccines is the development of anti-vector immunity, which can hinder the use of the same vectors for future vaccines. Nevertheless, viral vector vaccines remain a potential emergency consideration for pandemic outbreaks, as well as for highly contagious difficult-to-tackle pathogens alone or in combination with other emerging strategies [17].

While the lessons learned from the COVID-19 crisis helped us to better prepare for pandemic outbreaks by newly emerging viruses, a parallel downside that occurred is the reduction in access to routine immunization programs for other deadly drug-resistant pathogens, which re-emerge frequently. There are growing concerns about the re-emergence of measles [18], and immediate action is required to resume the health care services globally to their full potential. Intense research is required for improving the existing vaccines and developing novel ones for these pathogens. Bacillus calmette–guérin (BCG) vaccine, which is designed to protect against *Mycobacterium tuberculosis* (Mtb), is one of the most administered vaccines across the world. Although the incidence of tuberculosis has been drastically reduced by the routine BCG vaccination program in newborns, diseases caused by the other species of mycobacterium known as non-tuberculous mycobacteria (NTM) are still on the rise. They are less virulent than Mtb, but they are easily spread and develop drug resistance, leaving immunocompromised individuals at a greater risk [19]. The review article by Orujyan and colleagues discussed the potential of the BCG vaccine to impart partial protection against NTM, opening novel opportunities to design recombinant BCG vaccines, which can combat multiple mycobacterium species, including NTM [19].

Malaria caused by *plasmodium falciparum* is another disease that creates serious health issues and is a major cause of morbidity and mortality in tropical countries, for which the existing vaccines or drugs are not sufficiently useful. We have summarized the mechanisms of immunity generated in response to malaria infection and the available vaccines and vaccine adjuvants for malaria. The complex life cycle and different polymorphic stages of the malarial parasite are the biggest challenges that hinder the development of an effective vaccine for malaria. Although many antigen targets have been tested for malaria vaccine development, an ideal target has not been identified yet. We proposed the need to develop multivalent vaccines targeting different proteins presented in distinct stages of the parasite [20]. Besides, the currently used adjuvants are insufficiently effective, which warrants additional research to develop an ideal adjuvant formulation to help mitigate the global spread of malaria [20].

Overall, these six review articles published in the first edition have provided in-depth knowledge regarding recent developments in the field of vaccine research focusing on diverse topics and have given valuable insights and creative propositions on how to advance in tackling various existing challenges in the future.

In the 2nd edition of the special issue, Dang et al. developed a novel adjuvanted live-attenuated vaccine for human brucellosis, which is a zoonotic bacterial disease [21]. The existing live-attenuated *Brucella* vaccines cause reproductive health issues in cattle and were potentially pathogenic in humans due to the high dosage requirement. In this study, the authors have demonstrated that the use of Ag85a (a *Mycobacterium tuberculosis* cell wall derived diacylglycerol acyltransferase involved in lipid body formation) as an immune modulatory adjuvant significantly enhances CD4^+^ T and CD8^+^ T lymphocyte responses compared to vaccine alone. Knockout studies suggested that Ag85a achieves this protective immune response by activating the cyclic GMP–AMP synthase (cGAS)–stimulator of interferon genes (STING) signaling pathway and, hence, could be a solution to circumvent the high dose associated toxicity of live-attenuated *Brucella* vaccines in animals and humans [21].

The study carried out by Murtaza et al. tested the adjuvanticity of N-terminal bacterial flagellin fragments as a chimeric adjuvant with the truncated subunit protein (VP2) of the infectious bursal disease virus (IBDV) [22]. Flagellin is a well-established immunomodulatory adjuvant that activates toll-like receptor (TLR)5 and downstream MyD88 signalling pathways. The authors have demonstrated that two N-terminal fragments of *Salmonella typhimurium* flagellin, which contains the hotspot region for binding to TLR5, are sufficient to enhance the vaccine-induced humoral and cellular immune responses to IBDV. The results from this study demonstrated that the shorter fragments exhibit reduced pro-inflammatory responses compared to full-length flagellin C protein, whereas similar adjuvant property was maintained [22].

We hope that this 2nd edition of “Vaccines and Vaccine Adjuvants/Immunomodulators for Infectious Diseases” will continue to serve as a forum for creative scientific contributions and fruitful discussions from experts in the field.
